# Efficient Modeling of MS/MS Data for Metabolic Flux Analysis

**DOI:** 10.1371/journal.pone.0130213

**Published:** 2015-07-31

**Authors:** Naama Tepper, Tomer Shlomi

**Affiliations:** Department of Computer Science, Technion, Israel Institute of Technology, Haifa, Israel; Mayo Clinic, UNITED STATES

## Abstract

Metabolic flux analysis (MFA) is a widely used method for quantifying intracellular metabolic fluxes. It works by feeding cells with isotopic labeled nutrients, measuring metabolite isotopic labeling, and computationally interpreting the measured labeling data to estimate flux. Tandem mass-spectrometry (MS/MS) has been shown to be useful for MFA, providing positional isotopic labeling data. Specifically, MS/MS enables the measurement of a metabolite tandem mass-isotopomer distribution, representing the abundance in which certain parent and product fragments of a metabolite have different number of labeled atoms. However, a major limitation in using MFA with MS/MS data is the lack of a computationally efficient method for simulating such isotopic labeling data. Here, we describe the tandemer approach for efficiently computing metabolite tandem mass-isotopomer distributions in a metabolic network, given an estimation of metabolic fluxes. This approach can be used by MFA to find optimal metabolic fluxes, whose induced metabolite labeling patterns match tandem mass-isotopomer distributions measured by MS/MS. The tandemer approach is applied to simulate MS/MS data in a small-scale metabolic network model of mammalian methionine metabolism and in a large-scale metabolic network model of *E*. *coli*. It is shown to significantly improve the running time by between two to three orders of magnitude compared to the state-of-the-art, cumomers approach. We expect the tandemer approach to promote broader usage of MS/MS technology in metabolic flux analysis. Implementation is freely available at www.cs.technion.ac.il/~tomersh/methods.html

## Introduction

Metabolic flux analysis (MFA) is a method for quantifying in vivo metabolic fluxes that is commonly used to address problems in biotechnology and medicine [1–6]. It involves feeding cells with isotopic labeled nutrients (e.g. ^13^C labelled substrates), measuring metabolite isotopic labeling, and applying computational methods to estimate fluxes [1, 7–9].

MFA is based on the key observation that metabolite isotopic labeling patterns are uniquely determined by the distribtuion of metabolic flux in the network [10]. It is typically implemented as a non-convex optimization problem, searching for the most likely distribution of fluxes that would give rise to metabolite isotopic labeling that optimally matches experimental measurements. The running time of MFA methods becomes a major bottleneck when analyzing large-scale metabolic networks, consisting of hundreds of reactions, when repeatedly applied to compute accurate flux confidence intervals [11, 12], and in experimental design of isotopic labeling experiments [13]. The major factor that affects the performance of MFA implementations is the time required to simulate metabolite isotopic labeling for a candidate flux distribution.

A distinct labeling pattern of a certain metabolite is called an *isotopomer*, while the distribution of abundances of all isotopomers is reffered to as, *isotopomer distribution*. A metabolite with *n* carbons has *2* ^*n*^ distinct isotopomers; for example, as shown in Table 1, a metabolite having 4 carbons has 16 possible isotopomers. Previous studies have suggested the cumomers [14] and fluxomers [15] approaches for efficiently simulating the isotopomer distributions of all metabolites in a metabolic network given a flux vector. However, as the number of distinct isotopomers of a metabolite is exponentially dependent on the number of carbons that is has, these methods require a huge number of variables and may become computationally intractable.

**Table 1:** The distribution of isotopomers of metabolite A (shown in Fig 2) within tandemers of A, defined with respect to $A_{\left\{2,3\right\}}^{\left\{2,3,4\right\}}$.

Isotopomers	Tandemers
0000	[*M* + 0] > [*m* + 0]
0001	[*M* + 1] > [*m* + 0]
0010	[*M* + 1] > [*m* + 1]
0011	[*M* + 2] > [*m* + 1]
0100	[*M* + 1] > [*m* + 1]
0101	[*M* + 2] > [*m* + 1]
0110	[*M* + 2] > [*m* + 2]
0111	[*M* + 3] > [*m* + 2]
1000	[*M* + 0] > [*m* + 0]
1001	[*M* + 1] > [*m* + 0]
1010	[*M* + 1] > [*m* + 1]
1011	[*M* + 2] > [*m* + 1]
1100	[*M* + 1] > [*m* + 1]
1101	[*M* + 2] > [*m* + 1]
1110	[*M* + 2] > [*m* + 2]
1111	[*M* + 3] > [*m* + 2]

Isotopomers are represented by sequences of zeroes and ones, denoting non-labeled and labeled atoms, respectively.

Measuring the complete isotopomer distribution of metabolites is technically infeasible. Instead, mass-spectrometry is typically used to measure the relative abundance of a given metabolite having different number of labeled atoms (i.e. zero labeled atoms, one, two, etc). A set of isotopomers of a certain metabolite having the same mass is referred to as *mass-isotopomers*, while the relative abundance of mass-isotopomers denoted *mass-isotopomer distribution*. Notably, a mass-isotopomer distribution provides limited information on positional isotopic labeling, as isotopomers with the same number of labeled atoms have the same mass regardless of their position. Mass-isotopomer distributions can be calculated given the complete isotopomer distributions (by summing the abundances of isotopomers having the same number of labeled atoms). Alternatively, they can be directly and efficiently computed via the EMU approach [1].

Information on the positional labeling of metabolites can be obtained by tandem mass-spectrometry (i.e. MS/MS) and was previously shown to significantly improve quantification of metabolic fluxes via MFA [12, 16–18]. It works by isolating a single parent ion from the full spectrum and measuring its mass, followed by a collision that yields product ions whose mass is also measured. It can hence be employed to derive the mass-isotopomer distribution of a metabolite of interest and that of a collisional fragment. Most importantly, MS/MS can further measure the abundance of specific transitions from certain parent to product mass-isotopomers, referred to as *tandem mass-isotopomers* (also denoted here as *tandemers*, for short). We denote the number of labeled atoms of a parent ion by M+0 (having no labeled atoms), M+1 (having one labeled atom), etc., and the number of labeled atoms of a product ion by m+0, m+1, etc. We denote a transition from parent mass-isotopomer *M* + *i* to product mass-isotopomer *m* + *j*, by [*M* + *i*] > [*m* + *j*]. This provides additional information on positional labeling beyond that available via the mass-isotopomer distribution of the parent and product fragments separately (describing the abundance of a metabolite having various combinations of specific mass-isotopomers for the parent and product molecules). The relative abundance of all tandemers is referred to as a *tandem mass-isotopomer distribution* (or tandemer distribution, for short).

Currently, there is no method for efficiently simulating tandemer distributions. Previous applications of MFA given MS/MS data have inefficiently computed the complete isotopomer distributions for all metabolites in the network (for example, via cumomers [18]) in order to simulate tandemer distributions. Here, we present the tandemer method for efficiently simulating MS/MS measurements (i.e. tandemer distributions) of metabolites in a metabolic network. It builds upon and extends ideas set forward by the EMU method which efficiently simulates mass-isotopomer measurements [1].

## Theory

### A formal definition of tandemers

**Fig 1:**
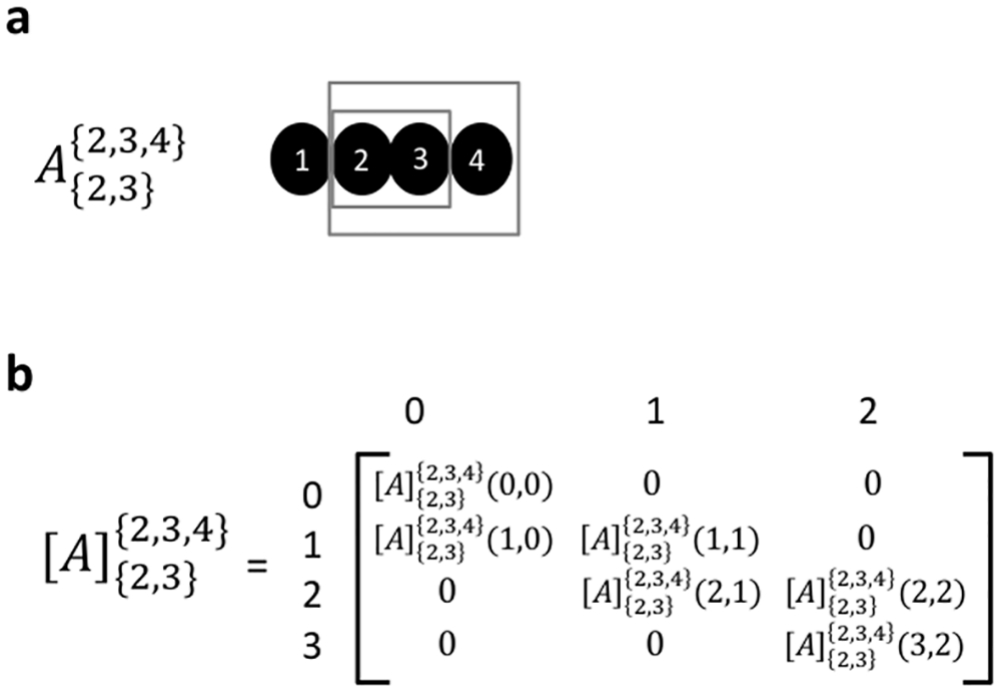
(a) Metabolite A and its MFP $A_{\left\{2,3\right\}}^{\left\{2,3,4\right\}}$ with parent fragment N = {2,3,4} and product fragment K = {2,3}. (b) The tandemer distribution matrix ${\left[A\right]}_{\left\{2,3\right\}}^{\left\{2,3,4\right\}}$. The abundance of infeasible tandemers in ${\left[A\right]}_{\left\{2,3\right\}}^{\left\{2,3,4\right\}}$ is, by definition, zero.

We denote a *metabolite fragment pair* (*MFP*) of a metabolite A with parent fragment N and product fragment K by $A_{K}^{N}$, with N ⊆ {1…n} (where n is the total number of atoms), and K ⊆ N. For example, Fig 1a shows metabolite A having 4 carbons and an associated MFP $A_{\left\{2,3\right\}}^{\left\{2,3,4\right\}}$, where N = {2,3,4} and K = {2,3}. A tandemer of metabolite A, [*M* + *i*] > [*m* + *j*] with respect to a MFP $A_{K}^{N}$ is defined as a set of isotopomers of A having 0 ≤ i ≤ |N| labeled atoms within the parent fragment N, and 0 ≤ j ≤ |K| labeled atoms within the product fragment K. A tandemer is considered feasible if it does not represent an empty set of isotopomers, i.e. when j is no larger than i (as the product fragment K is enclosed within the parent fragment N), and no smaller than i − (|N| − |K|) (when all atoms that are in the parent but not in the product fragment are labeled). The number of feasible tandemers for $A_{K}^{N}$ is hence (|N| − |K| + 1)(|K| + 1) [18].

The entire tandemer distribution of A, with respect to the MFP $A_{K}^{N}$, can be represented by a *tandemer distribution matrix* $[{A]}_{K}^{N}$ with |N| + 1 rows (representing the number of labeled atoms in the parent fragment; from zero to |N|), and |K| + 1 columns (representing the number of labeled atoms in the product fragment), such that $[{A]}_{K}^{N}$(i,j) is the relative abundance of tandemer [*M* + *i*] > [*m* + *j*]. As entries in $[{A]}_{K}^{N}$ represent distinct events whose sum is 1, the matrix represents a probability distribution. Notably, the abundance of infeasible tandemers is by definition zero. Fig 1b shows the tandemer distribution matrix $[{A]}_{\left\{2,3\right\}}^{\left\{2,3,4\right\}}$, while Table 1 shows the corresponding feasible tandemers. For example, given a metabolite A having 4 carbons, where the abundance of the isotopomer 1001 is 0.6, that of 0101 is 0.3 and 1011 is 0.1 (and the abundance of all other isotopomers of A is zero), the the tandemer distribution matrix of MFP $A_{\left\{2,3\right\}}^{\left\{2,3,4\right\}}$ is:

$${\left[A\right]}_{\left\{2,3\right\}}^{\left\{2,3,4\right\}}=\begin{array}{c} \begin{array}{c} 0 \end{array}\\ 1\\ \begin{array}{l} 2\\ 3 \end{array} \end{array}\begin{array}{c} \begin{array}{ccc} 0 & 1 & 2 \end{array}\\ \left[\begin{array}{c} \begin{array}{ccc} 0 & 0 & 0\\ 0.6 & 0 & 0\\ 0 & 0.4 & 0 \end{array}\\ \begin{array}{ccc} \;0\; & \;0\; & 0 \end{array} \end{array}\right] \end{array}$$

### Calculating metabolite tandemer distributions

**Fig 2:**
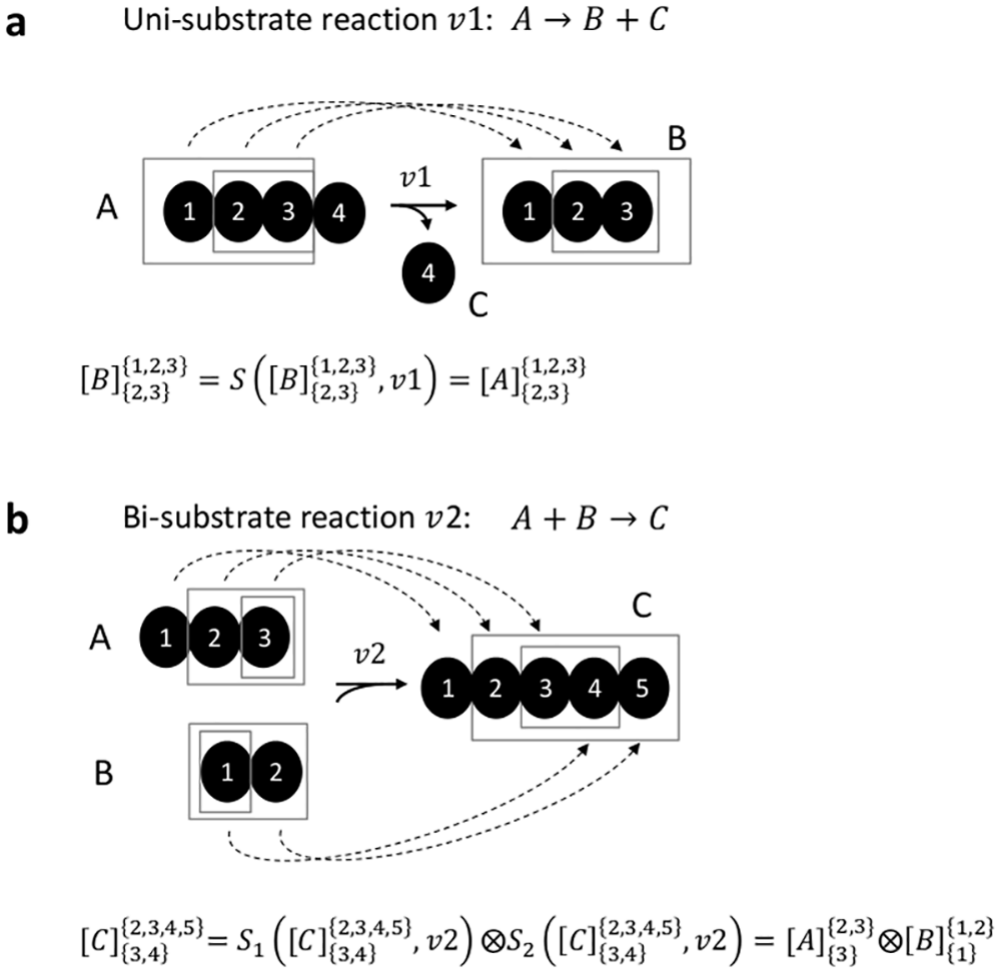
Calculating the tandemer distribution matrix of a product MFP based on tandemer distribution matrices of substrate MFPs in uni-substrate (a) and bi-substrate (b) reactions.

Under isotopic steady-state, for metabolite B that is produced solely through one biochemical reaction with a single substrate, A, the tandemer distribution matrix ${\left[B\right]}_{N}^{M}$ is equal to $[{A]}_{N^{'}}^{M^{'}}$, where atoms in M’ and N’ are mapped to atoms in M and N, respectively (Fig 2a). We refer to $A_{N^{'}}^{M^{'}}$ as the *substrate MFP* of $B_{N}^{M}$ via reaction i, and denote it by ${S(B}_{N}^{M},i).$

For metabolite C that is produced solely through one reaction with two substrates, A and B, $[{C]}_{N}^{M}$ can be calculated based on two tandemer distribution matrices: $[{A]}_{N_{1}}^{M_{1}}$ (where atoms in M_1_ and N_1_ are mapped to atoms in M and N, respectively) and ${\left[B\right]}_{N_{2}}^{M_{2}}$ (where atoms of M_2_ and N_2_ are similarly mapped to M and N; Fig 2b). $A_{N_{1}}^{M_{1}}$ and $B_{N_{2}}^{M_{2}}$ are further referred to as the substrate MFPs of $C_{N}^{M}$ via reaction i, and denote by ${S_{1}(C}_{N}^{M},i)$ and $S_{2}(C_{N}^{M},i)$ respectively. Specifically, $\left[{C]}_{N}^{M}(i,j\right)$ can be calculated as following:

$$\text{C}\left(\text{i},\text{j}\right)=\sum_{\begin{array}{c} 0\leq {\text{i}}_{1}<{\text{m}}_{1}\\ 0\leq {\text{i}}_{2}<{\text{m}}_{2}\\ {\text{i}}_{1}+{\text{i}}_{2}=\text{i} \end{array}}\sum_{\begin{array}{c} 0\leq {\text{j}}_{1}<{\text{n}}_{1}\\ 0\leq {\text{j}}_{2}<{\text{n}}_{2}\\ {\text{j}}_{1}+{\text{j}}_{2}=\text{j} \end{array}}\text{A}\left({\text{i}}_{1},{\text{j}}_{1}\right)\cdot \text{B}\left({\text{i}}_{2},{\text{j}}_{2}\right)$$ (1)

We refer to $[{C]}_{N}^{M}$ as being equal to the Cauchy product between matrices $A^{m_{1}\times n_{1}}$ and $B^{m_{2}\times n_{2}}$, denoted A⨂B (extending the definition of Cauchy product between vectors to matrices).

For example, let us consider the bi-substrate reaction shown in Fig 2b, in which A (having 3 carbons) is condensed with B (having 2 carbons) to make C, with carbons from A mapped to the first 3 carbons in C and atoms from B mapped to the last 2 carbons in C. In this case, the tandemer distribution matrix for $C_{\left\{3,4\right\}}^{\left\{2,3,4,5\right\}}$, can be calculated based on the Cauchy product of the tandemer distributions of the substrate MFP's $A_{\left\{3\right\}}^{\left\{2,3\right\}}$ and $B_{\left\{1\right\}}^{\left\{1,2\right\}}$.

Under isotopic steady-state, a tandemer distribution matrix for the MFP $A_{K}^{N}$ is determined based on the corresponding tandemer distributions of all of its substrate MFPs according to the following balance equation:

$${\left[A\right]}_{K}^{N}\sum_{i\in \left\{\begin{array}{c} reactions\\ producing\;A \end{array}\right\}}v_{i}\;=\;\sum_{i\in \left\{\begin{array}{c} Uni-substrate\\ reactions\\ producing\;A \end{array}\right\}}\left[S\left(A_{K}^{N},\;i\right)\right]\;\cdot \;v_{i}\;+\;\sum_{i\in \left\{\begin{array}{c} Bi-substrate\\ \;reactions\\ producing\;A \end{array}\right\}}\left[S_{1}\left(A_{K}^{N},\;i\right)\right]\;⊗\;S_{2}\left[\left(A_{K}^{N},\;i\right)\;\right]\;\cdot v_{i}$$ (2)

where *v*_*i*_ is the flux through reaction *i*.

### An algorithm for simulating tandemer distributions

**Fig 3:**
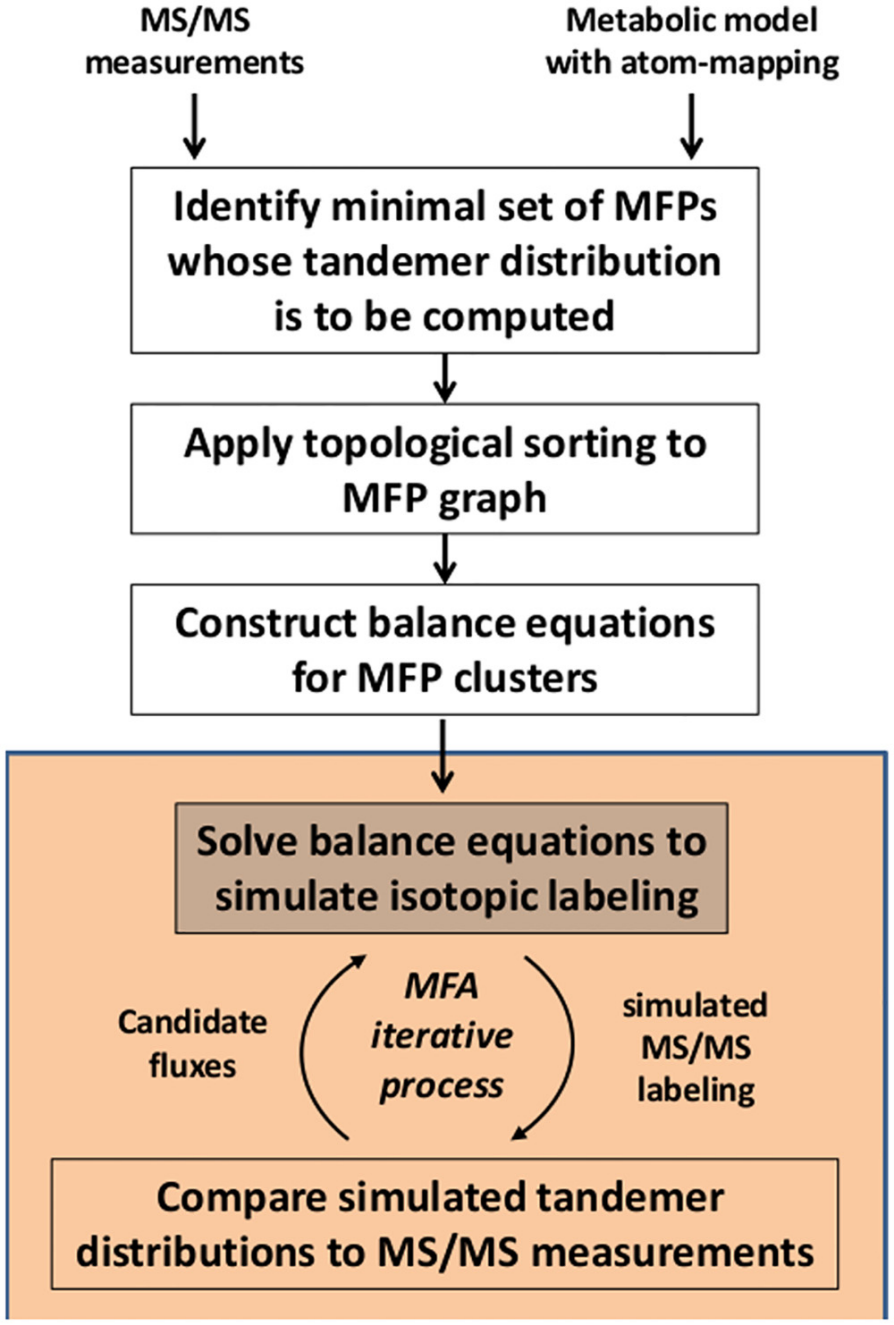
An outline of the tandemers approach. First, given MS/MS measurements and a metabolic model, a minimal set of MFPs is identified constructing an MFP graph. Second, MFPs are clustered and sorted and third, isotopic balance equations are formulated for each MFP cluster. Given a candidate flux vector, tandemer distributions are calculated by solving the set of isotopic balance equations.

Our goal is to efficiently simulate the tandemer distribution for a pre-defined set of metabolites (for which corresponding experimental data might be available). We assume that a metabolic network model with reaction atom-mappings (describing the mapping of substrate to product metabolite atoms in each reaction) and candidate fluxes are given. To address this problem, we present the tandemers approach, whose outline is shown in Fig 3. A detailed explanation of the various steps of the algorithms is provided below, while a Matlab implementation is available at: www.cs.technion.ac.il/~tomersh/methods.html.

#### 2.3.1. Identifying a minimal set of MFPs whose tandemer distributions are needed to simulate MS/MS measurements

The identification of a minimal set of MFP's in the metabolic network whose tandemer distributions would enable simulating the tandemer distribution of a given set of metabolites is done using a recursive procedure, in a similar manner to that presented in the EMU approach [1]. Specifically, starting with a list of MFPs for which tandemer distributions are available, we traverse the metabolic network to iteratively add substrate MFPs (via the definition of substrate MFPs provided above). This procedure ends when no more new MFPs can be added to the list.

The total number of MFPs depends on many factors, including the structure of the metabolic network, number of atoms per metabolite, reaction atom mappings, and number of metabolites for which experimental MS/MS data is available as input. In theory, for each metabolite with n carbons, the worst case number of possible MFPs could go up to:

$$\sum_{\text{i}=1}^{\text{n}}\sum_{\text{j}=1}^{\text{i}}\left({}_{\text{i}}^{\text{n}}\right)\left({}_{\text{j}}^{\text{i}}\right)<3^{n}$$ (3)

Hence, theoretically, the number of MFPs for a certain metabolite found by the recursive procedure described above may exceed the number of possible isotopomers for that metabolite (which is 2^n^). In practice, as we show below, applying this method on various metabolic networks, the number of MFPs found per metabolite is markedly lower than the number of isotopomers, resulting in improved running time compared to existing methods.

#### 2.3.2. MFP’s clustering and ordering

In a uni-substrate reaction producing MFP $B_{K}^{N}$, the size of the parent fragment in $B_{K}^{N}$ equals that of its substrate $A_{K'}^{N'}\;=\;S(B_{K}^{N},i)$ (i.e. |N’| = |N|), while in a bi-substrate reaction, the size of the parent fragment is larger than that of both its substrate MFPs (noting that a bi-substrate reaction in which one of the substrate MFP has a parent fragment of size zero can be regarded as a uni-substrate reaction). Hence, a tandemer distribution with parent fragment of a certain size is linearly dependent upon tandemer distributions with parent fragment of the same size, and non-linearly dependent (via Cauchy product) only upon tandemer distributions with smaller parent fragments (see Eq 2). Therefore, all tandemer distributions can be computed by sequentially solving linear balance equations for sets of tandemer distributions with increasing sizes (as Cauchy product of tandemer distribution matrices of smaller parent fragments can be computed before reaching tandemer distributions of larger parent fragments; similarly to the EMU [1] and cumomer [19] approaches).

Following a method proposed by [20], we divided the linear balance equations into smaller sets of equations that can be sequentially solved. Specifically, we construct a directed graph whose nodes represent the identified MFPs and edges connect an MFP $A_{K}^{N}$ with its substrate $S(A_{K}^{N},i)$ for uni-substrate reactions, and with both its substrates $S_{1}(A_{K}^{N},i)$ and $S_{2}(A_{K}^{N},i)$ for bi-substrate reactions. Notably, for a given MFP in this graph, its parent fragment size is not smaller than those of all MFPs having a directed edge towards it (see Fig 2). Hence, decomposing the graph into strongly connected components [21] and applying topological sorting on the identified clusters [22] leads to a cascade of MFP clusters, each consisting of MFPs having the same parent fragment size, with clusters ordered according to a non-decreasing fragment parent size (see Example below in Section 2.4). Iterating through the list of clusters, all tandemer distributions in a given cluster can be calculated via a set of linear equations based on tandemer distributions inferred in previous clusters (via Eq (2); where non-linear terms associated with bi-substrate reactions are calculated based on tandemer distributions inferred in previous clusters)[22].

#### 2.3.3. Formulating and solving a series of linear balance equations for MFPs in each cluster

Tandemer distributions for MFPs in a cluster are linearly dependent on each other, given the corresponding tandemer distributions for MFPs in previous clusters. Notably, the set of balance equations for tandemer distribution matrices for MFPs in the i’th cluster can be formulated as following:

$$\;\;\;A_{i}X_{i}=B_{i}Y_{i}$$ (4)

where *X*_*i*_ is a matrix whose rows represent the tandemer distributions for MFPs in the i’th cluster (i.e. each tandemer distribution represented as a row vector; removing infeasible tandemers), *Y*_*i*_ is a matrix whose rows represent tandemer distributions and Cauchy product of tandemer distributions computed for previous clusters, and *A*_*i*_ and *B*_*i*_ consist of corresponding fluxes.

Solving a set of balance equations for MFPs in the i’th cluster requires calculating the inverse of *A*_*i*_, whose number of rows (and columns) is equal to the number of MFPs in the cluster. As the cumomers approach also involves grouping cumomers in clusters and inverting flux matrices (similar to *A*_*i*_, here) whose size depends on the number of cumomers in each cluster, the running time of cumomers and tandemers approaches can be compared in terms of cumomers and MFPs cluster sizes, and the time needed to invert the corresponding flux matrices. Theoretically, an *n* fold reduction in the number of MFPs versus cumomers in a cluster should result in n^3^ improvement in running time. Notably, the number of non-feasible tandemers in each tandemer distribution matrix (represented by the number of column of *X*_*i*_ has a negligible effect on the time require to solve Eq (4), which is dominated by the n^3^ time required to calculate the inverse of *A*_*i*_).

### An example of the tandemers approach on a toy metabolic network

**Fig 4:**
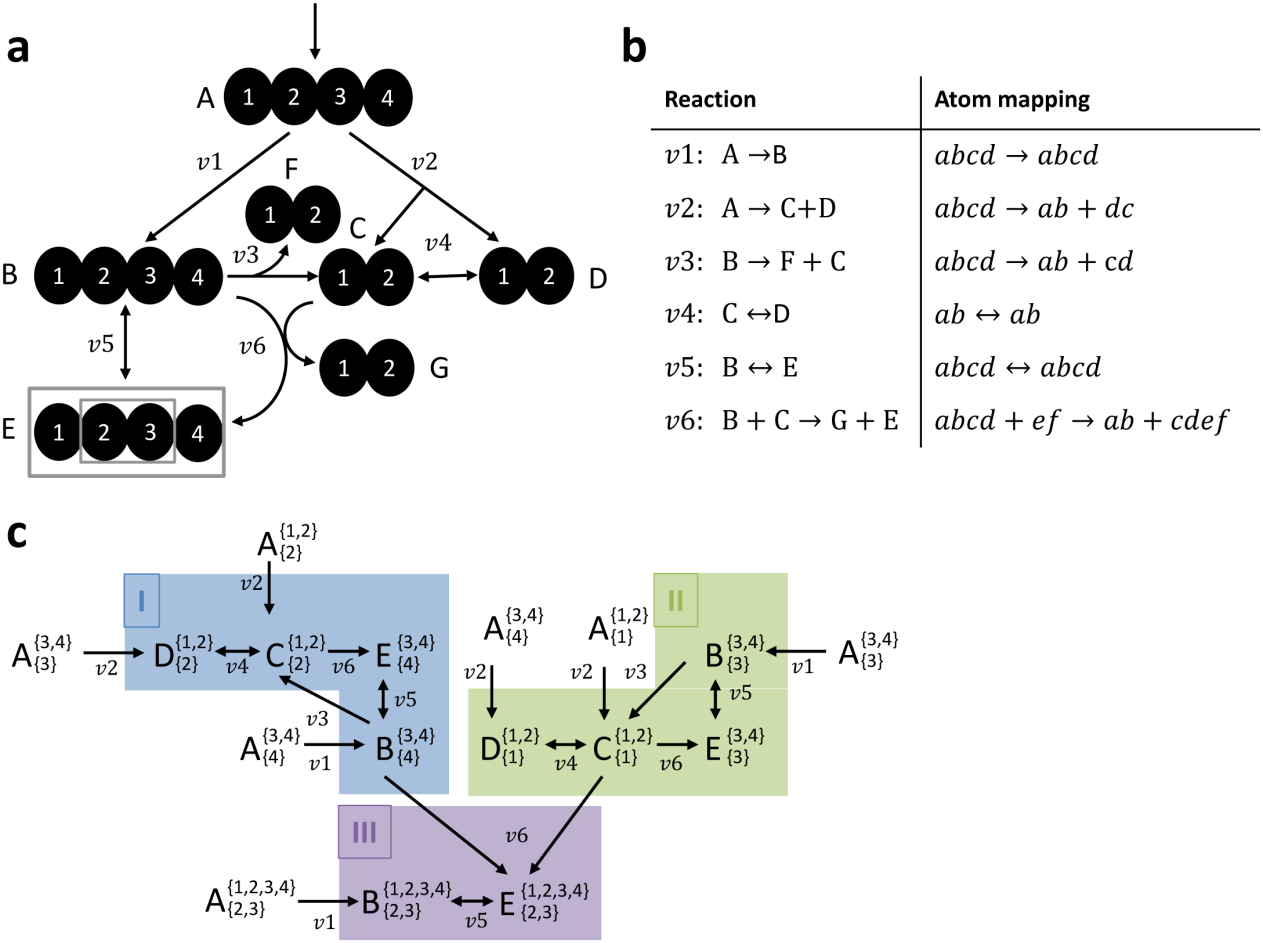
(a) A toy metabolic network, where the labeling pattern of A that is supplied in the media is assumed to be known, and the tandemer distribution of ${\left[E\right]}_{\left\{2,3\right\}}^{\left\{1,2,3,4\right\}}$ is to be calculated; (b) Atom mapping for network reactions; (c) An MFP graph and three strongly connected components whose numbering is determined via topological sorting.

In this section we describe the application of the tandemers approach on a toy metabolic network shown in Fig 4a, where atom mappings are given in Fig 4b. We assume that metabolic fluxes as well as the labeling pattern of A are known (considering that isotopically labeled A is given the growth media) and aim to compute the tandemer distribution of a $E_{\left\{2,3\right\}}^{\left\{1,2,3,4\right\}}$ (assumed to be measured via MS/MS). Applying the recursive procedure described in the previous section, starting from $E_{\left\{2,3\right\}}^{\left\{1,2,3,4\right\}}$, we identify a total of 10 MFPs (for metabolites other than A) whose tandemer distribution is needed to compute that of $E_{\left\{2,3\right\}}^{\left\{1,2,3,4\right\}}$. The MFP graph and its three connected components are shown in Fig 4c. Notably, cluster (III) depends on clusters (I) and (II), while the latter clusters are mutually independent.

For example, the isotopic balance equation for tandemer distribution matrices in cluster (I) is formulated as following, according to Eq (4):

$$\left[\begin{array}{c} -\left(v_{1}^{}+v_{5}^{b}\right)\\ v_{3}^{}\\ 0\\ v_{5}^{f} \end{array}\begin{array}{c} 0\\ -\left(v_{2}^{}+v_{3}^{}+v_{4}^{b}\right)\\ v_{4}^{f}\\ v_{6}^{} \end{array}\begin{array}{c} 0\\ v_{4}^{b}\\ -\left(v_{2}^{}+v_{4}^{f}\right)\\ 0 \end{array}\;\begin{array}{c} v_{5}^{b}\\ 0\\ 0\\ -\left(v_{5}^{f}+v_{6}^{}\right) \end{array}\right]\;\cdot \;\left[\begin{array}{cccc} {\left[B\right]}_{\left\{4\right\}}^{\left\{3,4\right\}}\left(0,0\right) & {\left[B\right]}_{\left\{4\right\}}^{\left\{3,4\right\}}\left(1,0\right) & {\left[B\right]}_{\left\{4\right\}}^{\left\{3,4\right\}}\left(1,1\right) & {\left[B\right]}_{\left\{4\right\}}^{\left\{3,4\right\}}\left(2,1\right)\\ {\left[C\right]}_{\left\{2\right\}}^{\left\{1,2\right\}}\left(0,0\right) & {\left[C\right]}_{\left\{2\right\}}^{\left\{1,2\right\}}\left(1,0\right) & {\left[C\right]}_{\left\{2\right\}}^{\left\{1,2\right\}}\left(1,1\right) & {\left[C\right]}_{\left\{2\right\}}^{\left\{1,2\right\}}\left(2,1\right)\\ {\left[D\right]}_{\left\{2\right\}}^{\left\{1,2\right\}}\left(0,0\right) & {\left[D\right]}_{\left\{2\right\}}^{\left\{1,2\right\}}\left(1,0\right) & {\left[D\right]}_{\left\{2\right\}}^{\left\{1,2\right\}}\left(1,1\right) & {\left[D\right]}_{\left\{2\right\}}^{\left\{1,2\right\}}\left(2,1\right)\\ {\left[E\right]}_{\left\{4\right\}}^{\left\{3,4\right\}}\left(0,0\right) & {\left[E\right]}_{\left\{4\right\}}^{\left\{3,4\right\}}\left(1,0\right) & {\left[E\right]}_{\left\{4\right\}}^{\left\{3,4\right\}}\left(1,1\right) & {\left[E\right]}_{\left\{4\right\}}^{\left\{3,4\right\}}\left(2,1\right) \end{array}\right]\;=\left[\begin{array}{ccc} -v_{1}^{} & 0 & 0\\ 0 & -v_{2}^{} & 0\\ 0 & 0 & -v_{2}^{}\\ 0 & 0 & 0 \end{array}\right]\;\cdot \;\left[\begin{array}{cccc} {\left[A\right]}_{\left\{4\right\}}^{\left\{3,4\right\}}\left(0,0\right) & {\left[A\right]}_{\left\{4\right\}}^{\left\{3,4\right\}}\left(1,0\right) & {\left[A\right]}_{\left\{4\right\}}^{\left\{3,4\right\}}\left(1,1\right) & {\left[A\right]}_{\left\{4\right\}}^{\left\{3,4\right\}}\left(2,1\right)\\ {\left[A\right]}_{\left\{2\right\}}^{\left\{1,2\right\}}\left(0,0\right) & {\left[A\right]}_{\left\{2\right\}}^{\left\{1,2\right\}}\left(1,0\right) & {\left[A\right]}_{\left\{2\right\}}^{\left\{1,2\right\}}\left(1,1\right) & {\left[A\right]}_{\left\{2\right\}}^{\left\{1,2\right\}}\left(2,1\right)\\ {\left[A\right]}_{\left\{3\right\}}^{\left\{3,4\right\}}\left(0,0\right) & {\left[A\right]}_{\left\{3\right\}}^{\left\{3,4\right\}}\left(1,0\right) & {\left[A\right]}_{\left\{3\right\}}^{\left\{3,4\right\}}\left(1,1\right) & {\left[A\right]}_{\left\{3\right\}}^{\left\{3,4\right\}}\left(2,1\right)\\ 0 & 0 & 0 & 0 \end{array}\right]$$

Considering that the tandemer distribution ${\left[B\right]}_{\left\{4\right\}}^{\left\{3,4\right\}}$ is calculated as part of cluster (I) and ${\left[C\right]}_{\left\{1\right\}}^{\left\{1,2\right\}}$ as part of cluster (II), enables to calculate the Cauchy product between the two matrices prior to solving the balance equations for cluster (III). Given the Cauchy product between ${\left[B\right]}_{\left\{4\right\}}^{\left\{3,4\right\}}$ and ${\left[C\right]}_{\left\{1\right\}}^{\left\{1,2\right\}}$, the isotopic balance equations for cluster (III) are linear:

$${\left[B\right]}_{\left\{2,3\right\}}^{\left\{1,2,3,4\right\}}∙(v_{1}^{}+v_{5}^{b})\;=\;{\left[A\right]}_{\left\{2,3\right\}}^{\left\{1,2,3,4\right\}}∙v_{1}^{}+{\left[E\right]}_{\left\{2,3\right\}}^{\left\{1,2,3,4\right\}}∙v_{5}^{b}$$

$${\left[E\right]}_{\left\{2,3\right\}}^{\left\{1,2,3,4\right\}}∙(v_{5}^{f}+v_{6}^{})\;=\;{\left[B\right]}_{\left\{2,3\right\}}^{\left\{1,2,3,4\right\}}∙v_{5}^{f}+{\left[B\right]}_{\left\{4\right\}}^{\left\{3,4\right\}}⨂{\left[C\right]}_{\left\{1\right\}}^{\left\{1,2\right\}}∙v_{6}^{}$$

## Results

### Applying the tandemers method on a small-scale model of mammalian methionine metabolism

**Fig 5:**
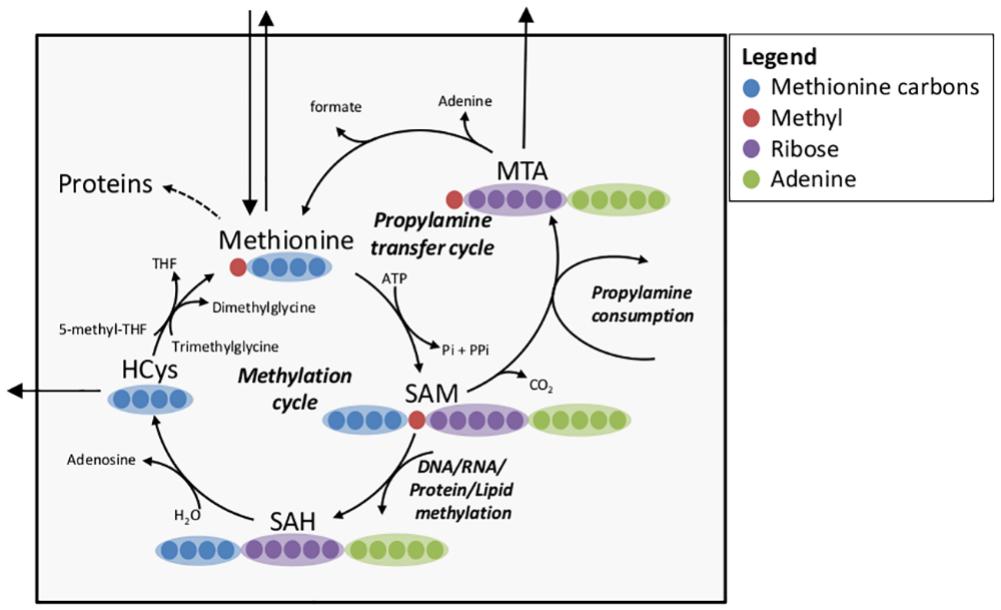
Methionine metabolism, including transmethylation cycle, polyamine biosynthesis and methionine salvage cycle. Metabolites abbreviations: SAM: S-Adenosylmethionine; SAH: S-Adenosylhomocysteine; HCyc: L-Homocysteine; MTA: Methylthioadenosine.

To demonstrate the applicability of the tandemers method for efficiently computing experimental MS/MS data in ^13^C labeling experiments, we applied it on a simplified metabolic network model of mammalian cellular metabolism of methionine (Fig 5, S1 File). Methionine metabolism involves two partially overlapping cyclic pathways for transmethylation (of protein and DNA) and propylamine transfer (for polyamine biosynthesis). The metabolic donor of both the methyl and propylamine groups is S-adenosylmethionine (SAM), which has 15 carbons (hence, a high-carbon metabolite). The product metabolites S-adenosylhomocysteine (SAH) and methylthioadenosine (MTA) also have high number of carbons, 14 and 11 carbons respectively (as one additional SAM carbon is oxidized to CO_2_ prior to the propylamine transfer). Considering that the number of isotopomers of a metabolite with n carbons is 2^n^, explicitly modeling the entire isotopomer distribution of all five metabolites in this network (as done in the cumomers method) would require 52,306 variables.

To apply the tandemers method, we utilized experimentally determined fluxes in this network as input [23]. We assume that the carbon labeling pattern of metabolites that are outside the scope of the model, including that of media methionine, ATP, and 5-methyl-tetrahydrofolate (i.e. the labeling of the methyl group in 5-methyl-THF) are known. First, we applied the tandemers method to compute the tandemer distribution of all 5 metabolites in the network, assuming that parent fragments are the intact metabolites, and that product fragments are the adenine group in SAM, SAH, and MTA, and the four methionine carbons other than the methyl group for methionine and HCys. The resulting number of MFPs whose tandemer distributions are found to be required to compute the tandemer distributions of the given MFPs is 35. These are divided into clusters, such that the largest cluster has only 4 MFPs. In comparison, applying the cumomers method given the same input data would require a total of 52,306 cumomers (the same as the number of isotopomers), divided into clusters whose largest one has 10,197 cumomers.

Next, we ran both the tandemers and cumomers methods multiple times, choosing a different subset of metabolites to calculate their tandemer distribution in each run. The parent fragment was assumed to be the intact metabolite and the product fragment was chosen to be the ribose, adenine, propylamine, or the four methionine carbons other than the methyl group. We find an average number of only 33 MFPs per run of the tandemers method, while the cumomers method requiring of 52,306 cumomer variables regardless of assumed input. The average running time of the tandemers method was found to be 0.00046 seconds, while that of the cumomers method was 0.68 seconds, ~1500-fold higher (Table 2). Notably, the significant reduction in running time will be especially important for designing optimal isotope tracing experiments, requiring numerous (many thousands for large-scale networks) repeated simulations of isotope labeling patterns for possible flux distributions [13].

**Table 2:** Comparison of the performance of the cumomers and tandemers methods in calculating tandemer distributions on mammalian methionine and *E*. *coli* networks.

	Mammalian methionine metabolism model			*E*. *coli* model		
	Variable count	Maximal cluster size	Running time	Variable count	Maximal cluster size	Running time
Cumomers	52,306	10,197	0.68	19,404	4,016	3.3
Tandemers	33	4	0.00046	695	32	0.01

### Applying the tandemers approach in a large-scale metabolic network model of *E*. *coli*

To further demonstrate the applicability of the tandemers approach, we applied it on a large-scale metabolic network model of *E*. *coli* [13]. This isotopomer model accounts for glycolysis, TCA cycle, pentose phosphate pathway, oxidative phosphorylation, pyruvate metabolism, anaplerotic reactions and other central and biosynthetic pathways, with a total of 206 metabolites and 405 reactions. Notably, metabolites with a high number of carbons were not included in this network reconstruction to facilitate the application of the cumomers approach (by lumping surrounding reactions together) [13]. The total number of isotopomers in this large-scale network is hence surprisingly low, reaching 19,404 isotopomers (much lower than that in the small-scale methionine network applied above). Hence, even though this network model is substantially larger than the methionine network, we do not expect the tandemers approach to demonstrate the same level of improvement compared to the cumomers method.

We applied the tandemers method 1000 times to compute the tandemer distribution of randomly chosen sets of metabolites (having between 1 to 20 metabolites). The average number of resulting MFPs was 695, with a maximal MFP cluster size of 32. In comparison, applying the cumomers approach resulted in 19,404 cumomers, and a maximal cluster size of 4,016. The average running time of the tandemers and cumomers methods are 0.01 and 3.3 seconds, respectively, representing a ~300-fold improvement by the tandemers method (Table 2).

For the tandemers methods, the number of variables represents the number of MFP whose tandemer distribution is calculated; for the cumomers approach, it represents the number of cumomers. The number of these variables corresponds to the size of the flux matrices whose inverse is calculated by each method, and is hence proportional to overall running time (see Section 2.3.3). We report the average variable count, clustersize, and running time for the cumomers and tandemers methods in multiple simulations given different sets of metabolites and collisional fragments, as described above. Notably, considering that MFA applications and especially experimental design of isotope tracing experiments require thousands of repeated simulations of metabolite isotopic labeling, the ~1500-fold and 300-fold improvement in running time observed in the mammalian methionine network and on the *E*. *coli* network, respectively, is of a major practical importance.

## Discussion

Tandem MS holds great promise for metabolic flux analysis as it provides information on metabolite positional labeling [12, 16–18]. However, a major limitation in using MFA with MS/MS data is the lack of a computationally efficient method for simulating isotopic labeling data measurable via MS/MS. State-of-the-art methods such as cumomers that enable to simulate MS/MS data requires simulating the abundance of all distinct isotopomers, whose number is exponentially dependent on the number of atoms in each metabolite. Here, we described the tandemers approach that is specifically designed for efficiently computing tandem mass-isotopomer distributions measurable via MS/MS, demonstrating a roughly two to three orders of magnitude improvement in running time compared to the cumomers approach. The tandemers approach is especially useful when analyzing metabolic networks with metabolites having a high number of carbons, where modeling the entire isotopomer distribution may become computationally intractable.

In our application of the tandemers method on a metabolic network of mammalian methionine metabolism and for *E*. *coli*, we computed tandemer distributions for MFPs in which the parent fragment was the entire metabolite. This represents the case where no in-source fragmentation occurs during MS ionization, which is typically the situation with LC-MS. However, in-source fragmentation can occur (mostly with GC-MS), leading to measurement of tandemer distributions with parent fragment that is not the entire metabolite. Such in-source fragmentation can provide further useful information on positional labeling, for example, as was recently used to infer all distinct isotopomers of aspartate [24]. Obviously, the tandemers approach may also be used to compute tandemer distributions for MFPs with parent fragments that are not the entire metabolite.

In a recent study, we described a method, Metabolic Flux Analysis/Unknown Fragments (MFA/UF), capable of using MS/MS data to improve flux inference even when the positional origin of fragments is unknown [12]. MFA/UF extends upon standard MFA and jointly searches for the most likely metabolic fluxes together with the most plausible position of collisional fragments that would optimally match measured MS/MS data. To simulate MS/MS data given candidate fluxes and candidate collisional fragments, MFA/UF utilized the cumomers approach to simulate MS/MS labeling data. Considering that the tandemers approach was shown here to outperform the cumomers method, integrating it within MFA/UF is expected to lead to a significant improvement in running time.

Considering that a major current complication in utilizing MS/MS data in metabolic flux analysis involves the lack of computationally efficient methods for simulating such experimental measurements, we expect the tandemers approach to promote broader usage of this technology.

## Supporting Information

S1 File Methionine metabolism model. (XLSX)
