# Correction: Efficient Modeling of MS/MS Data for Metabolic Flux Analysis

**DOI:** 10.1371/journal.pone.0138630

**Published:** 2015-09-14

**Authors:** Naama Tepper, Tomer Shlomi

The following information is missing from the Funding section: This work was supported by the European Union Seventh Programme for Research, Technological Development and Demonstration under the grant agreement STREPSYNTH (project No. 613877). The funders had no role in study design, data collection and analysis, decision to publish, or preparation of the manuscript.
